# Predicting biomarkers for ovarian cancer using gene-expression microarrays

**DOI:** 10.1038/sj.bjc.6601603

**Published:** 2004-02-03

**Authors:** T R Adib, S Henderson, C Perrett, D Hewitt, D Bourmpoulia, J Ledermann, C Boshoff

**Affiliations:** 1 Cancer Research UK Viral Oncology Group, Wolfson Institute for Biomedical Research, University College London, Cruciform Building, Gower Street, London WC1E 6BT, UK; 2 Department of Obstetrics and Gynaecology, University College London, Cruciform Building, Gower Street, London WC1E 6BT, UK; 3 Department of Oncology, University College London, London WC1E 6BT, UK

**Keywords:** Ovarian cancer, gene expression microarrays, biomakers, MGB2

## Abstract

Ovarian cancer has the highest mortality rate of gynaecological cancers. This is partly due to the lack of effective screening markers. Here, we used oligonucleotide microarrays complementary to ∼12 000 genes to establish a gene-expression microarray (GEM) profile for normal ovarian tissue, as compared to stage III ovarian serous adenocarcinoma and omental metastases from the same individuals. We found that the GEM profiles of the primary and secondary tumours from the same individuals were essentially alike, reflecting the fact that these tumours had already metastasised and acquired the metastatic phenotype. We have identified a novel biomarker, mammaglobin-2 (MGB2), which is highly expressed specific to ovarian cancer. MGB2, in combination with other putative markers identified here, could have the potential for screening.

Ovarian cancer is the leading cause of death from gynaecological malignancy, with an estimated 24 000 and 6800 new cases in the US and UK, respectively, during 2001 (Greenlee *et al*, 2001; Swerdlow *et al*, 2001). Early-stage disease is largely asymptomatic, and most patients are diagnosed when disease has spread beyond the pelvis with an associated 5-year survival of less than 20% (Ozols, 2002). This is partly due to the lack of reliable screening strategies. While around 90% of women with advanced disease have elevated serum CA125, this marker alone is neither sufficiently sensitive nor specific for use as a screening tool (Menon and Jacobs, 2001). Despite new cytotoxic regimens, survival has remained largely unchanged over the past 20 years. Identification of new molecular signatures of early disease is a key goal of ovarian cancer research.

Gene-expression microarray (GEM) profiles have previously been used to compare the expression profile of ovarian cancer with that of the normal ovary (Ono *et al*, 2000; Welsh *et al*, 2001). We extended this approach by using a more extensive set of probes (Affymetrix U95Av2), and also characterised metastatic disease in a search for molecular markers of progression. We investigated the potential specificity of a number of putative biomarkers by examining their expression in a panel of other epithelial tissues and tumours.

## MATERIALS AND METHODS

### Ovarian tissue samples

Four snap-frozen normal ovarian samples, and six pairs of primary and omental serous adenocarcinoma (Stage IIIC) from the same individuals were collected at the time of surgery at the University College Hospitals NHS Trust. The six paired samples of primary and secondary ovarian cancer were taken at the time of primary surgery prior to chemotherapeutic intervention. The normal ovarian samples were taken at the time of surgery for benign disease. H&E-stained sections were examined and verified histopathologically to be stage III serous adenocarcinomas. All samples comprised at least 70% tumour, except one omental sample which had 5% tumour content. The normal ovarian samples were verified to be free of any pathology, including benign cysts. Ovarian epithelium was macrodissected from the underlying stroma, and was used for subsequent analysis. For the real-time quantitative RT–PCR data, we used, in addition, three serous tumours of low malignant potential (LMP). All patients gave preoperative informed consent, and the study was approved by the ethics committee of the Royal Free and University College Medical School.

### RNA sample preparation

Tissue specimens were homogenised in lysis buffer using a rotary homogeniser. Total RNA was extracted using the Qiagen RNeasy® kit (Qiagen, Valencia, CA, USA), according to the manufacturer's instructions. The integrity of the RNA was assessed by ethidium bromide staining after agarose gel electrophoresis. Total RNA (20 *μ*g) was used to synthesise double-stranded cDNA using the Superscript® Choice System (Life Technologies), with the template being used for an *in vitro* transcription reaction to yield biotin-labelled antisense cRNA (BioArray™ High Yield RNA Transcript Labelling Kit, Enzo Diagnostics, Farmingdale, NY, USA). Fragmentation, hybridisation and scanning were performed according to the Affymetrix GeneChip® protocol, using the U95Av2 oligonucleotide microarrays containing ∼12 000 genes (Affymetrix, Santa Clara, CA, USA).

### Real-time quantitative RT–PCR

Four genes, shown in the microarray system to be significantly upregulated, were selected for analysis with real-time quantitative reverse transcription–polymerase chain reaction (qRT–PCR). Primer pairs for each gene were designed using the Primer Express® Software (Applied Biosystems) and selected to have the same annealing temperature (60°C). The primer sequences used were: mammaglobin B2 (MGB2), forward 5′-CCGCTGCAGAGGCTATGG-3′, reverse 5′-CATCAGTCCAAAGTTTTTCAGAGTTCT-3′, kallikrein 6 (KLK6), forward 5′-GCGGACCCTGCGACAAG-3′, reverse 5′-GGATAAGGACCCCACCACAGA-3′; serum amyloid A1 (SAA1), forward 5′-TTCTCACGGGCCTGGTTTT-3′, reverse 5′-GCCTCGCCAAGGAACGA-3′ and hepsin (HPN), forward 5′-GGCTCGAGTCCCCATAATCAG-3′, reverse 5′-GGTAGCCAGCACAGAACATCTTG-3′. Primers were tested by conventional PCR and the PCR products were sequenced prior to real-time quantitation to confirm the specificity (data not shown). Primer optimisation and efficiencies were performed prior to the relative quantitation of the expression of the genes (data not shown). Real-time qRT–PCR was performed on an ABI PRISM® 7000 SEQUENCE DETECTOR (Applied Biosystems, Applera UK, Cheshire, UK) using the SYBR® Green PCR Master Mix (Applied Biosystems) in duplicate, with triplicate nontemplate controls (NTC) in a 25 *μ*l PCR reaction. cDNA (1 *μ*l) was used in a 25 *μ*l PCR mixture containing 1 × SYBR® Green PCR mix (Applied Biosystems) and 0.3 *μ*M of each primer for all genes, apart from HPN where 0.6 *μ*M forward and reverse were used. The cDNAs were amplified by denaturation for 10 min at 95°C, followed by 40 cycles of denaturation at 95°C for 15 sec and annealing extension at 60°C for 1 min. The threshold cycle (*C*_T_), which represents the PCR cycle at which an increase in reporter fluorescence above a baseline signal can first be detected, was calculated as previously described (Heid *et al*, 1996). The relative expression of each gene was determined on the basis of the *C*_T_ value. The housekeeping gene GAPDH was used to normalise the quantity of cDNA used. Average GAPDH *C*_T_ value was subtracted from that of each target gene to obtain a Δ*C*_T_ value, that is, normalised target gene expression relative to GAPDH. An average Δ*C*_T_ value was obtained for each of the five groups of 19 cDNA ovarian samples (normal: *n*=5, LMP: *n*=3, primary: *n*=5 and metastasis: *n*=2). Each average Δ*C*_T_ was also subtracted from that of a calibrator (average Δ*C*_T_ value of all the normal samples which provide the physiological expression of each gene target) to give the ΔΔ*C*_T_ value, that is, normalised target gene expression in the different groups relative to normal. Since *C*_T_ values are measured when PCR amplification is still in the exponential phase, the relative quantitative value can be expressed as 2^−ΔΔ*C*T^, as 2 corresponds to the PCR product doubling in each cycle in the exponential phase.

### Immunohistochemistry (IHC)

IHC was performed for hepsin (HPN) on 30 formalin-fixed, paraffin-embedded tissues histologically characterised into three distinct tissue groups: normal ovarian, primary ovarian serous cystadenocarcinoma and metastatic (omentum), to confirm expression at the protein level. Sections were cut at 4 *μ*m, deparaffinised and rehydrated in a series of graded alcohols, before being heated in a microwave in Tris-EDTA (TE) for 25 min. Endogenous peroxidase activity was blocked by 10 min incubation with 0.5% hydrogen peroxide (H_2_O_2_) in methanol, prior to the application of goat polyclonal primary antibody (1 : 50; Santa Cruz Biotechnology Inc., Insight Biotechnology Ltd, Wembley, UK) for 1 h at 22°C. A biotinylated, anti-goat secondary antibody (1 : 400; DAKO, Cambridgeshire, UK) was applied for 30 min, after which slides were incubated with the streptavidin-peroxidase complex (DAKO) for a further 30 min. Sections were visualised by application of diaminobenzidine (DAB) substrate (DAKO) for 7 min, followed by a wash in running H_2_O and counterstaining for 2 min with Mayer's haematoxylin (DAKO). All sections were then dipped in acid alcohol to remove excess haematoxylin, and immediately placed in running H_2_O. After dehydration in graded alcohols, slides ended in xylene, and were mounted in DPX.

### Data analyses

Background subtraction, normalisation and expression values of our data were calculated using the rma algorithm (Irizarry *et al*, 2003), available as part of the Affymetrix package of the Bioconductor open-source software library for the statistical language R (http://www.bioconductor.org). The rma algorithm differs from the standard Affymetrix algorithm in a number of ways; most importantly, the data are quantile–quantile normalised at the probe level, prior to calculation of a final expression summary from the positive match (or PM) probes alone. This algorithm improves measurement precision, reducing the variation between replicate data, particularly of low-expressed genes. Differential expression was calculated using the Benjamini–Hochberg step-down false-discovery rate (FDR) algorithm set to 0.05, implemented using the Bioconductor multtest package. This algorithm adjusts *P*-values upwards to discount the effects of multiple testing. It is a less-conservative adjustment (admitting more errors) than the more common, but here impractically conservative, Bonferroni or Holm algorithms.

### Comparative GEM data

Publicly available GEM data from normal epithelia-rich tissues were obtained from the Genomics Institute of the Novartis Research Foundation expression atlas (http://expression.gnf.org). Prostate and lung adenocarcinoma data were obtained from the Whitehead Institute Centre for Genomic Research (http://www-genome.wi.mit.edu/c gi-bin/cancer). Both data sets were in the original Affymetrix CEL format, and were normalised and analysed using the same methods as our own data described above.

## RESULTS

Four normal ovarian samples, plus six paired primary (stage IIIC) and secondary samples from the same individual were analysed. The histopathology of adjacent sections showed that 70–90% of primary samples and 90% of metastases (except one sample) constituted tumour cells. The normal ovarian samples were verified to be free from any benign pathology. Differences in gene expression discussed below were all tested for significance using a FDR of 0.05, using the Benjamini–Hochberg step-down algorithm (Benjamini and Hochberg, 1995) (see Materials and methods). For clarity, gene names and abbreviations used throughout the text are summarised in Table 1

**Table 1:** Summary of the names and abbreviations of genes discussed

**Symbol**	**Probe-id**	**Acc ID**	**Full name**
ADN	40282_2_at	M84256	Adipsin
AGRN	33454_at	AF016903	Agrin
BF	35822_at	L15702	B-factor
CD24	266_at	L33930	CD24 antigen
CD9	39389_at	L33690	CD9 antigen
CLDN3	33904_at	AB000714	Claudin 3
CLDN4	35276_at	AB000712	Claudin 4
CP	39008_at	M13699	Ceruloplasmin
EZH2	37305_at	U61145	Enhancer of zeste homologue 2
FHL2	38422_at	U29332	Four-and-a-half LIM domains 2
FOXO1A	40570_at	AF032885	Forkhead box O1A
HE4	33933_at	X63187	WAP four-disulphide core domain 2
HPN	37639_at	X07732	Hepsin
IFI-15K	1107_at	M13755	Interferon, alpha-inducible protein, 15 kDa
IGL	33273_at	X57809	Immunoglobulin lambda locus
KLK10	36838_at	AF055481	Kallikrein 10
KLK11	40035_at	AB012917	Kallikrein 11
KLK13	36406_at	AA401397	Kallikrein 13
KLK2	217_at	S39329	Kallikrein 2
KLK3	1804_at	X07730	Kallikrein 3
KLK6	37554_at	U62801	Kallikrein 6
KLK7	38143_at	L33404	Kallikrein 7
KLK8	37131_at	AB008390	Kallikrein 8
KLK18	35766_at	M26326	Keratin 18
KRT19	40899_at	Y00503	Keratin 19
KRT8	33824_at	X74929	Keratin 8
LMNB1	37985_at	L37747	Lamin B1
LPL	41209_at	M15856	Lipoprotein lipase
LU	40093_at	X83425	Lutheran blood group
OP	2092_at	J04675	Osteopontin
OPCML	41093_at	AF070577	Opioid-binding protein/cell adhesion molecule like
PEG3	39701_at	AB006625	Paternally expressed 3
PLIN	37122_at	AB005293	Perilipin
PRAME	157_at	U65011	Preferentially expressed antigen of melanoma
PRSS8	634_at	L41351	Protease, serine 8
PTTG1	40412_at	AF095288	Pituitary tumour transforming 1
SAA1	33272_at	AA829286	Serum amyloid A1
SCGB2A1	41066_at	NM_002407	Secretoglobin, family 2A, member 1
SLP1	32275_at	X04470	Secretory leucoctye protease inhibitor
TACSTD2	575_s_at	J04152	Tumour-associated calcium signal transducer 2
VEGF	36100_at	AF024710	Vascular endothelial growth factor
WISP2	35898_at	AF100780	WNT1-inducible signalling pathway protein 2

The symbol here is the most commonly used abbreviation, usually from Online Mendelian Inheritance in Man (OMIM). Probe is the unique Affymetrix probe ID. The Accession is the NCBI Refseq, or reference sequence for this gene.

.

### Primary ovarian disease

**Figure 1:**
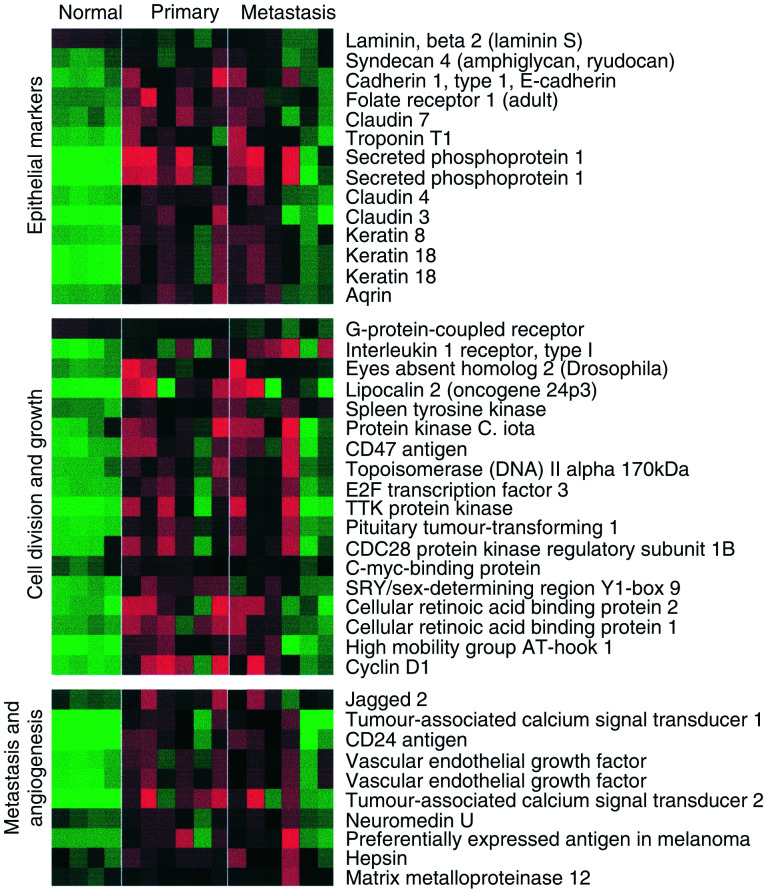
Heatmap showing genes upregulated in serious ovarian primary and omental metastatic tumours compared to the normal ovary. Columns represent individual tissue samples; rows represent individual genes. Red and green cells represent transcript levels for each gene across the samples above and below the median, respectively. All differences are significant at the *P*<0.05 level after multiple testing adjustment (see Materials and methods).

**Figure 2:**
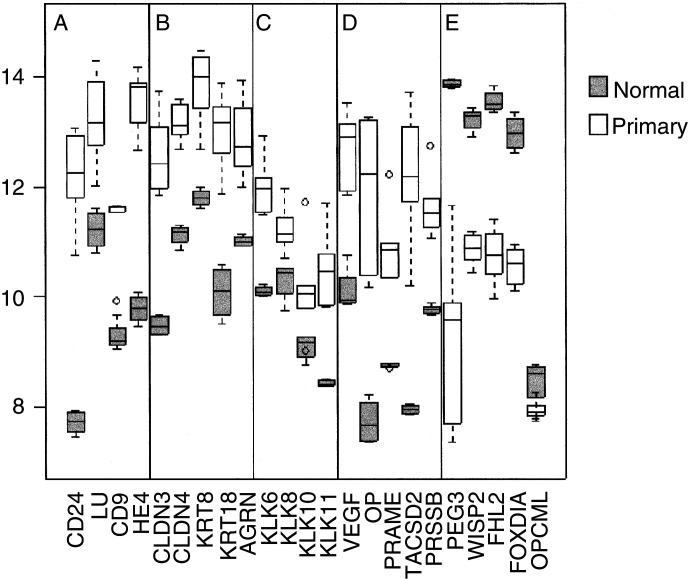
Box and whisker plots show expression of selected genes in both normal (shaded, *n*=4) and primary tissues (unshaded, *n*=6). The selected genes are split into five categories (**A**–**E**) from left to right: (**A**) for comparison with previous ovarian cancer GEM studies, (**B**) epithelial markers, (**C**) kallikrein serine protease family, (**D**) a selection of previously described serious ovarian cancer markers and (**E**) genes with loss of expression in primary tumours. Box and whisker plots show a central median line, an interquartile box. Whiskers 1.5 times the interquartile range, and outliers of these, are shown as circles.

There were 421 genes more than two-fold and 118 genes more than three-fold overexpressed in primary compared to normal tissue. Figure 1

shows significantly overexpressed genes in primary ovarian cancer sorted into functional groups. These groups include genes associated with epithelia and cell–cell contact, such as secreted phosphoprotein 1 (osteopontin, OP), folate receptor 1, claudins 3 and 4 (CLDN3, 4), keratins 8, 18 and 19 (KRT8, 18, 19), and agrin (AGRN). These are also shown in Figure 2B

, and could reflect the epithelial origin of these tumours. Genes involved in cell division and growth include cyclin D1, cellular retinoic acid-binding protein 2 and lipocalin 2 (oncogene 24p3). Metastasis and angiogenesis genes include jagged 2, tumour-associated calcium signal transducer 2 (TACSTD2), vascular endothelial growth factor (VEGF), CD24 antigen and neuromedin U.

We compared the consistency of our data with that of another study (Welsh *et al*, 2001), where overexpression of tumour genes in cancer were ranked according to a combined metric, using normal ovary as a baseline. The four genes CD24, WAP four-disulphide core domain 2 (HE4), CD9 and Lutheran blood group (LU) were found to be the most highly expressed by their method, and are also highly overexpressed in our own data set (Figure 2A). Where the data sets overlap, they are highly consistent.

We found that a number of kallikreins (KLKs), a family of trypsin-like serine proteases that include prostate-specific antigen (PSA/KLK3), were overexpressed in ovarian cancer. Kallikreins are being investigated as potential serum markers for adenocarcinomas such as prostate (KLK2), breast (KLK10, 12, 13) and ovary (KLK6, 8, 10, 11) (Diamandis and Yousef, 2002) (Figure 2C). In addition, we identified KLK7 as overexpressed in ovarian cancer.

We identified a number of overexpressed genes previously associated with ovarian and other cancers including VEGF, osteopontin (OP) (Kim *et al*, 2002), preferentially expressed antigen in melanoma (PRAME) (Steinbach *et al*, 2002), TACSD2 (or GA733-1) (Shetye *et al*, 1989; Szala *et al*, 1990) and prostasin (PRSS8) (Mok *et al*, 2001) (Figure 2D). These may all play a role in ovarian carcinogenesis.

**Figure 3:**
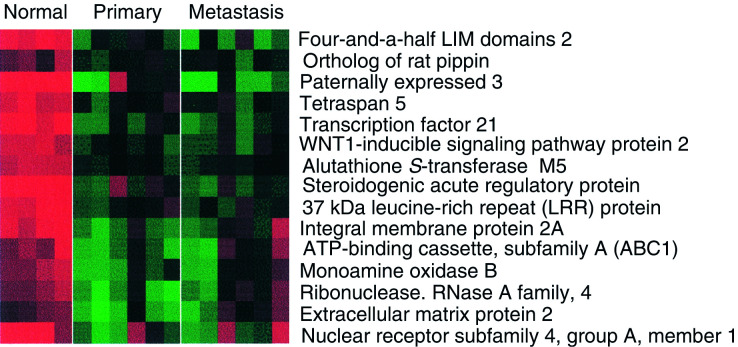
Genes downregulated in primary and secondary serous ovarian cancer compared to the normal ovary. All differences are significant at the *P*<0.05 level after multiple testing adjustment (see Materials and methods).

We identified 172 genes that were three-fold downregulated in primary ovarian cancer compared to the normal ovary (Figure 3

). Among these were putative tumour suppressors including the p53 mediator paternally expressed gene-3 (PEG-3) (Relaix *et al*, 1998; Deng and Wu, 2000), wnt-inducible signalling protein-2 (WISP-2), a member of the connective tissue growth factor family (Pennica *et al*, 1998), and the Rho-associated transcriptional coactivator four-and-a-half LIM domains 2 (FHL2) (Muller *et al*, 2002). However, the recently reported putative tumour suppressor in ovarian cancer, opioid-binding protein (OPCML), did not appear to have significant loss of expression in any of the samples studied here (Sellar *et al*, 2003) (Figure 2E).

### Omental metastasis

**Figure 4:**
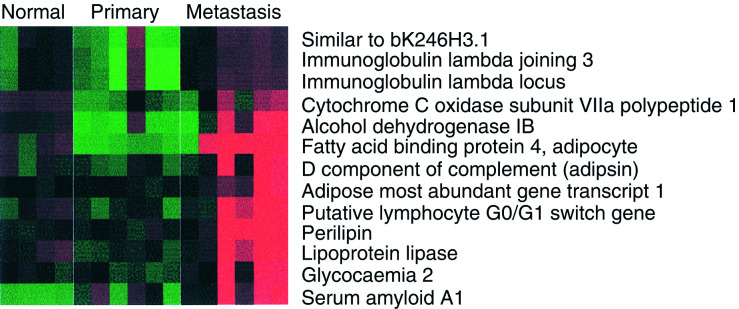
Genes upregulated in omental metastasis relative to normal ovary and primary ovarian cancer. The predominance of genes associated with adipocytes reflects the omental background. All differences are significant at the *P*<0.05 level after multiple testing adjustment (see Materials and methods).

**Figure 5:**
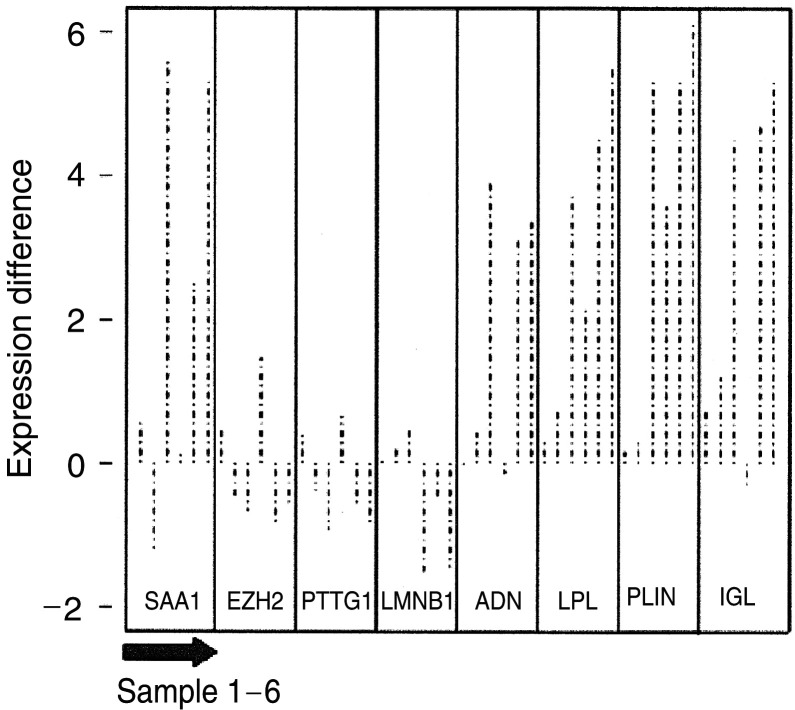
Expression of genes in metastatic and primary ovarian cancer samples (*n*=12, six-paired). The log difference of selected genes between the paired metastatic and primary ovarian cancer samples is plotted (metastatic: primary), so that upwards is higher in metastasis and downwards is lower. The paired *P*-values were SAA1-0.03, EZH2-0.82, PTTG1-0.47, LMNB1-0.41, ADN-0.04, LPL-0.01, PLIN-0.01 and IGL-0.01.

**Figure 6:**
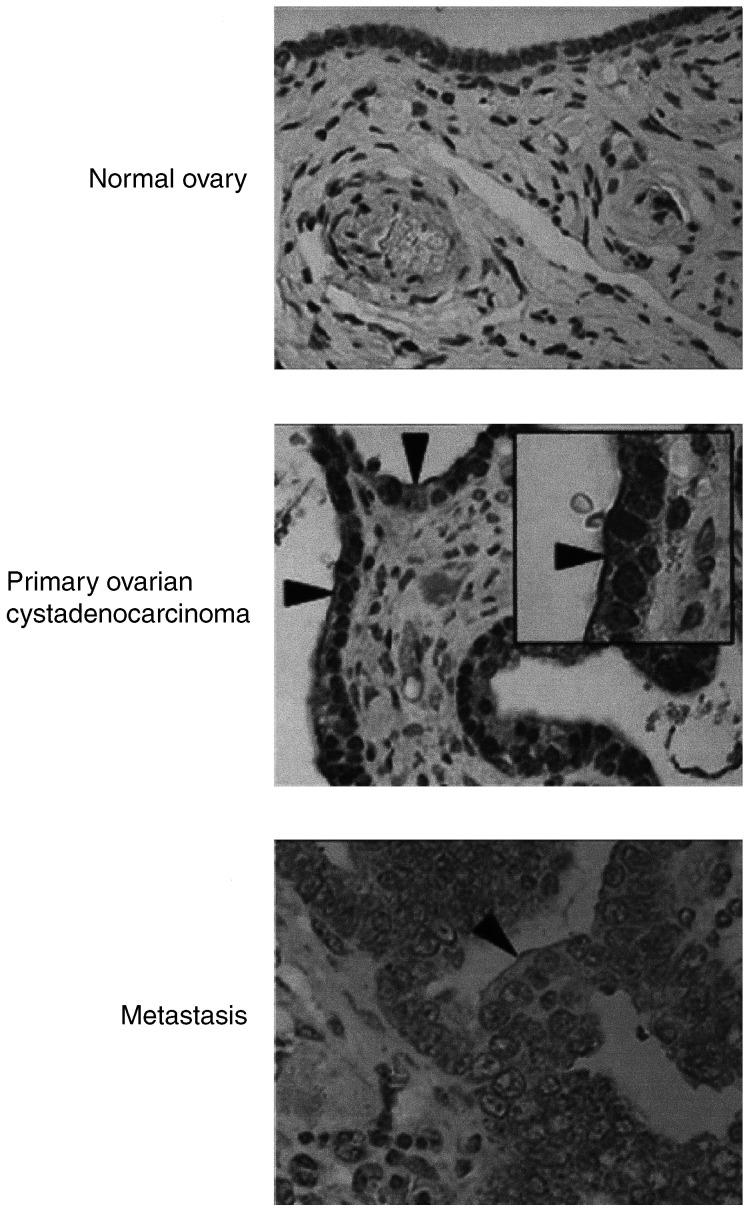
Immunohistochemical staining for hepsin. Hepsin stained normal and malignant epithelial cells. However, a prominent membrane staining (arrowheads) was only seen in malignant epithelial cells. Pictures × 40; inset × 100.

While there were 300 genes with more than three-fold difference between normal and primary samples, there were only 35 equally large differences between primary and omental metastases, all greater in metastases. These genes fell into two main groups. These included serum amyloid A1 (SAA1), which is a marker of inflammation and immunoglobulin (Ig) lamda-locus, which may reflect leucocyte infiltration. We found that many of the gene differences between primary and paired omental samples reflect the high adipocyte content in the omentum, such as adipsin, lipoprotein lipase and perilipin (Figure 4

). We found a number of putative invasion and metastasis predictive genes including enhancer of zeste homolog 2 (EZH2) (prostate cancer) (Varambally *et al*, 2002), pituitary tumour-transforming 1 interacting protein (PTTG1) and Lamin B1 (LMNB1) (adenocarcinoma) (Ramaswamy *et al*, 2003) to be unchanged in primary and omental specimens (Figure 5

). Essentially, the malignant primary and epithelial tumour are alike. Hepsin, a prostate cancer serum biomarker, while marginally overexpressed in primary, was further overexpressed in secondary ovarian cancer tissue. Immunohistochemistry for hepsin showed staining of both the normal ovarian surface epithelium (OSE) and malignant epithelial cells in primary and omental metastasis. The pattern in malignant cells was distinct, however, being localised to the membrane (Figure 6

).

### Validation of array data with qRT–PCR

**Figure 7:**
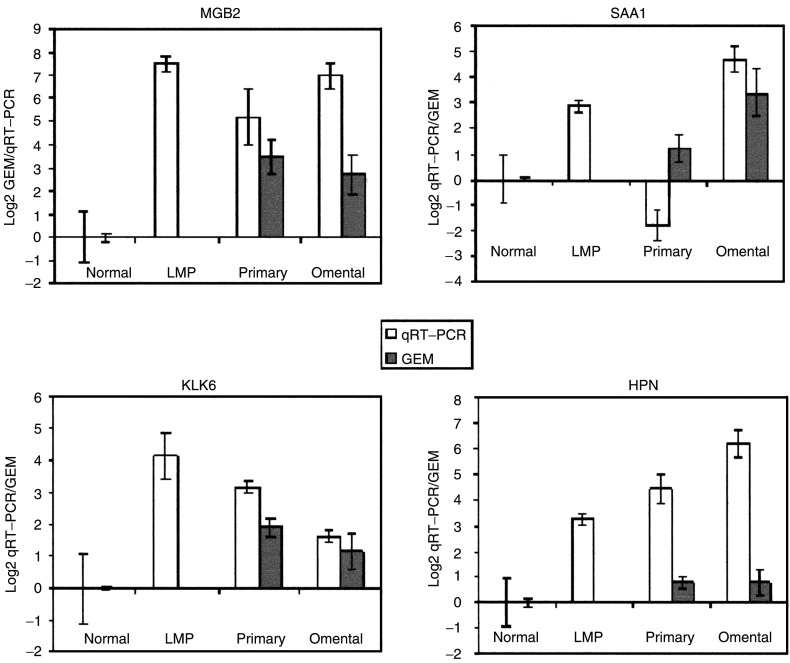
Comparison of qRT–PCR (clear bars, normal (*n*=5), primary (*n*=5), LMP (*n*=3) and metastasis (*n*=2)) and GEM data (shaded bars, normal (*n*=4), primary (*n*=6) and metastasis (*n*=6)) for MGB2, SAA1, KLK6 and HPN in normal, primary and omental metastasis samples. Gene-expression microarray data are in original Log2 scale, and qRT–PCR is single Log2 unit per round of amplification, error bars show the standard deviation. The normal level is taken as a 0 baseline reference for both.

In order to validate the gene-expression levels from the microarray experiments, we performed real-time qRT–PCR with GAPDH as a control in five normal ovaries, three LMP ovarian serous cancers, five primary ovarian serous cystadenocarcinomas and two omental metastases. Figure 3 shows the corresponding gene expression patterns of four genes: mammaglobin B2 (MGB2), serum amyloid A1 (SAA1), kallikrein-6 (KLK6) and hepsin (HPN) for normal ovary, primary and secondary disease on the microarrays, compared to that on qRT–PCR. Figure 7

demonstrates that the differential expression pattern and the quantitative expression level of each of these four genes, as determined by qRT–PCR, were comparable to those observed with the microarrays, confirming the reliability of our array expression data. Notably, qRT–PCR showed high expression of MGB2 and KLK6 in the LMP samples.

### New biomarkers

**Figure 8:**
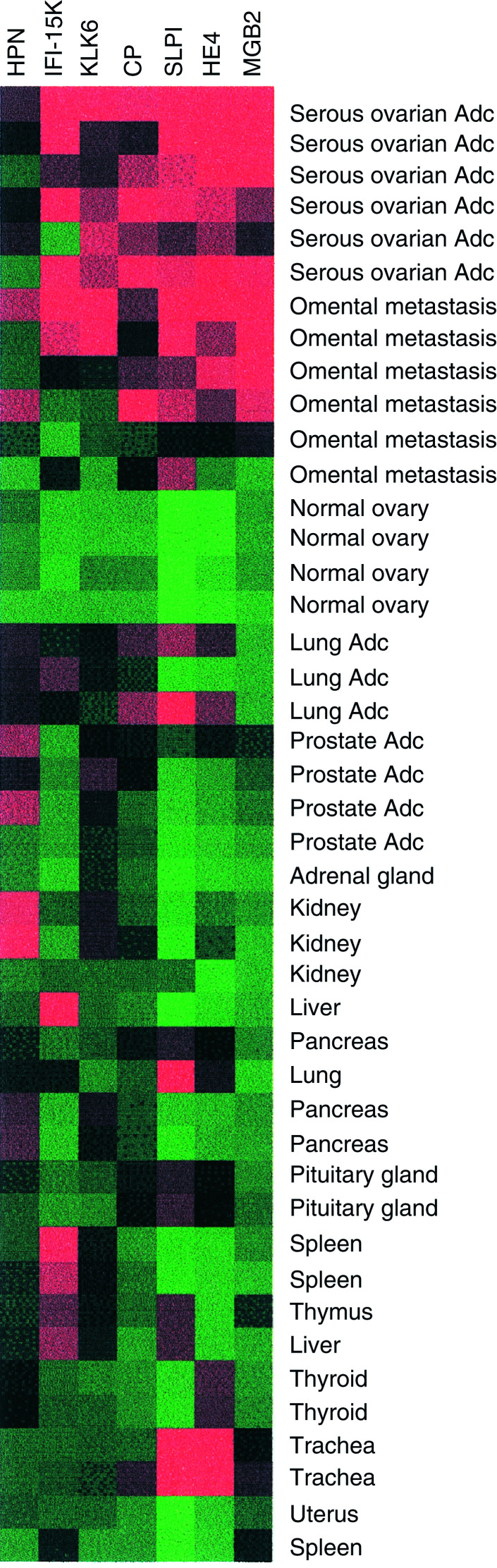
Gene-expression profile of putative biomarker MGB2 in ovarian serous adenocarcinoma and a panel of other tissues. Comparison with six previously described biomarkers HPN, IF1-15K, KLK6, CP, SLPI and HE4. Serious ovarian AdC=primary serous ovarian adenocarcinoma, omental metastasis=serious ovarian omental metastasis, lung AdC=lung adenocarcinoma, prostate AdC=prostate adenocarcinoma. Adrenal gland, kidney, liver, pancreas, pituitary gland, lung, spleen, thyroid, trachea and uterus, all represent the corresponding normal tissue specimens.

We identified a potential new biomarker MGB2 with: (a) higher expression in both primary and metastatic samples compared to the normal ovary, (b) high gross expression above the 80th percentile of all genes in primary and metastatic samples and (c) with high homology (58% amino-acid identity) to the known serum marker MGB. Figure 8

shows the GEM profile of MGB2 compared to that of six other proteins that have been suggested as potential biomarkers: HPN (Tanimoto *et al*, 1997), IFI-15K, KLK6 (Diamandis and Yousef, 2002), CP (Hough *et al*, 2001), SLPI (Shigemasa *et al*, 2001) and HE4 (Schummer *et al*, 1999) across a panel of epithelia-rich tumours and tissues. This panel was comprised of publicly available Affymetrix data from (see Materials and methods, Data analysis): (a) prostate adenocarcinoma (Singh *et al*, 2002), (b) lung adenocarcinoma (Bhattacharjee *et al*, 2001) and (c) the GNF gene expression atlas containing various primary epithelial tissues (Su *et al*, 2002). MGB2 in particular is specific to ovarian adenocarcinoma.

## DISCUSSION

We have used oligonucleotide microarrays representing ∼12 000 genes to investigate the GEM profiles of epithelial ovarian cancer. A number of groups have previously investigated gene-expression profiling of ovarian cancer using microarrays (Wang *et al*, 1999; Ismail *et al*, 2000; Ono *et al*, 2000; Mok *et al*, 2001; Shridhar *et al*, 2001; Welsh *et al*, 2001). These studies have focused on either the identification of gene products which can serve as ovarian cancer-specific markers (Mok *et al*, 2001), or on the initiation and progression of ovarian cancer (Ismail *et al*, 2000; Shridhar *et al*, 2001). This has been achieved by comparing the normal ovarian epithelium with ovarian cancer samples, as the majority of ovarian cancers are thought to arise from the ovarian surface epithelium, which exists as a single layer of cells covering the ovaries. This layer of cells easily sloughs off at the time of surgery by manual handling, and it is a challenge to obtain enough cells for use in any experimental procedures. Researchers have overcome this problem by firstly using short-term cell culture to increase the number of cells available (Ismail *et al*, 2000), secondly by RNA amplification (Ono *et al*, 2000) and thirdly by using commercially available RNA (Welsh *et al*, 2001). These approaches, however, have drawbacks: (i) short-term culture favouring the growth of only a subset of epithelial cells, (ii) RNA amplification leading to unequal amplification of all RNA transcripts in the cell population and (iii) the inclusion of a stromal component in commercially available RNA.

In this study, we used macrodissected epithelium from the normal ovarian tissue in addition to matched primary and secondary metastatic serous ovarian adenocarcinomas. Tumour specimens were verified histopathologically in five cases to comprise at least 70% tumour. We confirmed a number of ovarian cancer genes previously identified by GEM, for example, CD24 (Welsh *et al*, 2001), HE4 (Schummer *et al*, 1999), PRAME (Ismail *et al*, 2000), B-factor (properdin) (Shridhar *et al*, 2001), and, where our studies overlap, the data are highly consistent, despite the difference in methodology.

A large number of genes overexpressed in primary tumours were associated with epithelia. This might reflect the epithelial origin of these tumours or a transformed phenotype. HPN, for example, was marginally overexpressed in both primary and secondary ovarian cancer tissue, compared to the normal ovary (approx. two-fold). HPN is a serine protease that has been shown to be overexpressed in prostate cancer cells, and significantly correlates with poor clinical outcome (Dhanasekaran *et al*, 2001). We investigated hepsin further by performing IHC, and found the staining to be localised to the epithelial cells, suggesting that it may be a marker of epithelia rather than of malignancy (Figure 6). However, there was a notable difference in the pattern with malignant cells showing a distinct membranous staining, suggestive of heightened secretion.

We found few differences in the gene signature of stage III primary serous ovarian adenocarcinomas and their corresponding omental metastases. Various studies have shown that metastatic signatures within primary tumours are predictive of subsequent metastasis. We found that, within the stage III serous ovarian adenocarcinomas, a number of predictive genes including EZH2 (Varambally *et al*, 2002), PTTN and Lamin-B (Ramaswamy *et al*, 2003) are overexpressed in primary, at least as highly as in omental metastases (Figure 4). This supports the notion that most tumour cells in advanced primary ovarian lesions have acquired the genetic signature enabling invasion and metastasis. A GEM study comparing stage Ia (no ascites) with Ic (ascites, that is, metastatic spread) might identify genes that infer the propensity of ovarian tumour cells to metastasise, although it would be challenging to obtain sufficient material.

We identified a potential new biomarker MGB2, which is significantly overexpressed in primary and metastatic ovarian cancer compared to the normal ovarian tissue. This gene is part of the uteroglobin family, and is also overexpressed in endometrioid endometrial carcinomas (Moreno-Bueno *et al*, 2003), and the axillary lymph nodes of metastatic breast cancers (Ooka *et al*, 2000). A preliminary qRT–PCR analysis of MGB2 confirmed this finding and further demonstrated high expression in LMP samples (*n*=3). LMP tumours are a distinct subtype of epithelial ovarian cancer thought to be as an intermediate stage between clearly benign and malignant tumours. No biomarker to date is sufficiently specific for screening and monitoring disease progression in LMP tumours. MGB2 warrants further investigation in this subgroup.

The only widely used ovarian cancer marker CA125 lacks specificity (CA125 or MUC16 is not present on the U95Av2 array). Within the panel of data available to us, MGB2 appears to be a specific biomarker for ovarian tumours with low expression in most normal epithelial tissues and prostate and lung tumours. This survey was far from exhaustive, relying on available published GEM data. The screening and selection of candidates for further serological study will benefit from more publicly available data, in particular breast cancer. The recent development of multiplex techniques to screen sera for combinations of biomarkers shows promise for cancer screening (Petricoin *et al*, 2002). A combination of biomarkers including MGB2 rather than a single biomarker alone is more likely to give a specific signature for epithelial ovarian carcinoma. Our study demonstrates that GEM studies are a practical and economical prelude to streamline candidate genes for larger serological studies.
